# Health related quality of life in chronic kidney disease; a descriptive study in a rural Sri Lankan community affected by chronic kidney disease

**DOI:** 10.1186/s12955-020-01369-1

**Published:** 2020-04-23

**Authors:** Sameera Senanayake, Nalika Gunawardena, Paba Palihawadana, Shanika Senanayake, R. Karunarathna, Priyantha Kumara, Sanjeewa Kularatna

**Affiliations:** 1 Epidemiology Unit, Ministry of Health, Colombo, Sri Lanka; 2 World Health Organization, Colombo, Sri Lanka; 3 Australian Centre for Health Service Innovation, Queensland University of Technology, Brisbane, Australia; 4 North Central Provincial Directors Office, Anuradhapura, Sri Lanka

**Keywords:** Chronic kidney disease, Quality of life, Sri Lanka, Chronic kidney disease of unknown etiology

## Abstract

**Introduction:**

The current epidemic of chronic kidney disease (CKD) in Sri Lanka is ascribed to the exponential increase in the number of CKD patients, which cannot be attributed to any known etiology (CKDu). The aim of this study is to describe the health related quality of life (HRQOL) and the associated factors among CKD/CKDu patients in a rural district in Sri Lanka.

**Methods:**

A community based cross-sectional study included 1174 CKD/CKDu patients. Kidney Disease Quality of Life-Short Form was used to assess the HRQOL, while Centre for Epidemiologic Studies Depression Scale, General Health Questionnaire (GHQ) 12 and CKD Symptom Index – Sri Lanka were used to assess presence of depression, psychological distress and symptom burden respectively. Three summary scores; kidney disease (KDSC), physical (PCS) and mental (MCS) are derived from Kidney Disease Quality of Life-Short Form (KDQOL-SF™).

**Results:**

Mean age of the study population was 58.3 years (standard deviation (SD) 10.7). Median KDSC (58.4; inter-quartile range (IQR) 54.2–63.4), was higher than the median scores of PCS (35.0; IQR 26.2–41.9) and MCS (58.4; IQR 54.2–63.4). Multiple linear regression revealed low income, advanced stages of CKD, symptom burden, being positive for depression and psychological distress were significantly associated with low HRQOL.

**Conclusion:**

The HRQOL of the CKD patients in this rural Sri Lankan population was found to be poor. Superior socio-economic status, less physical and psychological symptom burden were found to be independently associated with better HRQOL. Periodical screening of the CKD patients for depression and psychological distress and measures to alleviate symptom burden seem to be important to improve the HRQOL of these patients.

## Introduction

Interest in the concept of health related quality of life (HRQOL) began in the early seventies and has increased over the last 20 years, becoming one of the central goals of patient management. HRQOL is not a universal phenomenon and there is no single definition to describe all its facets. World Health Organization Quality of Life Group (WHOQOL) in 1994 defined quality of life (QOL) as “an individual’s perception of their position in life in the context of culture and value systems in which they live and in relation to their goals, expectations, standards and concerns” [1]. However, HRQOL refers to QOL in the context of disease and health. Health is considered a multidimensional concept, thus, HRQOL is also multidimensional and incorporates domains related to physical, mental and emotional, and social functioning [2]. Therefore HRQOL is a subjective phenomenon usually influenced by expectations, person’s experience and beliefs [3].

Over the years, chronic kidney disease (CKD) has emerged as a major public health problem in Sri Lanka. Exact prevalence estimates of CKD are not available in Sri Lanka. Medical statistics on hospitalizations and deaths in the state sector hospitals in the country do not present data for CKD but present data on diseases of the urinary system. These statistics indicate that in 2017 diseases of the urinary system was the 6th leading cause of hospitalization in the country accounting up to 4.7% of total hospital admissions. Diseases of the urinary system was also the 8th leading cause of hospital deaths with a rate of 12.9 per 100,000 population [4]. The corresponding figure for the year 2012 was 12.1 per 100,000 population [5].

Chronic kidney disease is a chronic disease condition and by its progressive and disabling nature, has a significant impact on HRQOL of affected individuals [6]. From the early stages of the disease to its end stage, disabling symptoms, various food and restrictions to social life, fluid restrictions and associated stigma and taboos, affect daily life of the patients. Furthermore, CKD affects the mental well-being which in turn affects the HRQOL of patients. A recent study among CKD patients in Sri Lanka revealed that 75.0% (95% CI 72.5–77.5) of participants were psychologically distressed while 65.2% (95%CI 62.4–68.0) were depressed [7]. World over, the importance of including HRQOL indicators in the clinical management of CKD patients has been highlighted [8–11]. This has come to the limelight after several studies demonstrated the strong relationship between reduced HRQOL and increased morbidity and mortality [8–11]. Kidney Disease Outcomes Quality Initiative (K/DOQI), which is the leading organization in developing standards and guidelines pertaining to management of CKD, advises the HRQOL to be evaluated regularly in order to establish baseline function and monitor changes occurring over time as well as to evaluate the effects of various interventions in all patients with glomerular filtration rate (GFR) below 60 ml/min (stage III) [12]. Further, the Center for Medicare Services in United States has made it a mandatory requisite of routine measurement of HRQOL of all dialysis patients [3].

Chronic kidney disease has become a major health problem in the North Central Province (NCP) of Sri Lanka during the past two decades and this is mainly due to CKD which cannot be attributed to any known etiology [13]. This form of CKD is usually termed Chronic Kidney Disease of uncertain etiology (CKDu). There are records of an exponential increase in the number of cases of CKDu in the NCP since the early 1990’s [14]. Though the accurate proportions of known and unknown origin are not available, diseases of the urinary system, which includes CKD, is the leading cause of hospital deaths and the 5th leading cause of hospital admissions in NCP [15]. Research evidence indicates that the CKDu affected patients are mostly young and belong to the low socio-economic class [16]. Research evidence also confirm that the increase in burden of CKD in the past two decades has crippled this community leading to significant physical, psychological and economic hardships [17].

Systematic evaluation of the HRQOL of the CKD/CKDu patients in the rural districts of NCP has not been carried out to date. This study aims at assessing the disease related quality of life and the associated factors among CKD patients in the Anuradhapura district, a rural district in NCP. Evaluation of HRQOL among CKD/CKDu patients in this community can add new insight into the management of the disease as it allows the quantification of the disease consequences according to the patient’s perception and enables adjustment of medical decisions to their physical, emotional, and social needs. Furthermore, understanding the extent to which QOL is affected in patients with CKD, will help facilitate the implementation and evaluation of interventions which favorably impact the wellbeing of the patients.

## Methods

### Patient selection

A population based descriptive cross-sectional study was conducted in the district of Anuradhapura in the North Central Province (NCP) of Sri Lanka. Confirmed CKD patients more than 18 years of age with documented evidence of CKD, living in the Anuradhapura district were included in the study. The study period was from January 2016 to March 2016. Following patients were excluded from the study; patients with previous renal transplantation, critically ill patients and patients who were unable to provide rational information for any reason (e.g.; mental retardation). Informed consent was obtained from the eligible study participants prior to data collection.

The country is divided into 338 preventive healthcare areas, called Medical Officer of Health (MOH) areas. Anuradhapura district has 19 Medical Officer of Health areas and the study was conducted in all the 19 MOH areas. The number of patients to be included from each Medical Officer of Health area was based on probability proportionate to the size of CKD patients registered in each of the Medical Officer of Health area. Simple random sampling method was used to select the required number of participants from each Medical Officer of Health area. The population based CKD register, available at the Provincial Director of Health Services, records the patients with a confirmed diagnosis of CKD from renal clinics in hospitals of the NCP since 2003. This register was used as the sampling frame.

### Assessment of HRQOL

Health related quality of life was assessed using the locally validated Kidney Disease Quality Of Life-Short Form (KDQOL-SF™) version 1.3. The instrument was confirmed as having a good construct validity and test re-test reliability [18].

KDQOL-SF has two components; Kidney Disease Specific Component and SF-36. Of the total 81 questions in 19 domains, 43 questions assess 11 kidney disease specific components of HRQOL and SF-36 questionnaire in which the 36 questions assess the general health related HRQOL in eight domains.

The 11 domains of Kidney Disease Specific Component are: symptom/problem list (12 items), effects of kidney disease (8 items), burden of kidney disease (4 items), cognitive function (3 items), quality of social interaction (3 items), sexual function (2 items), sleep (4 items), social support (2 items), work status (2 items), patient satisfaction (1 item), and dialysis staff encouragement (2 items). SF-36 includes 36 items that measure eight domains and the eight domains are: physical function (10 items), role limitations caused by physical problems (4 items), role limitations caused by emotional problems (3 items), pain (2 items), general health perceptions (5 items), social function (2 items), emotional well-being (5 items), and energy/fatigue (4 items). The final item, the overall health rate item, asks the respondents to rate their health on a 0–10 response scale. Different questions have different answer options, which range from two to seven. When scoring, each question is scored in a scale ranging from 0 (worst health) to 100 (best health). All items in a domain are summed up and averaged to give an average score for each domain which ranges from 0 (worst health) to 100 (best health). Three summary scores; kidney disease summary component (KDSC), physical component summary (PCS) and mental component summary (MCS) are derived from the 19 domain scores of KDQOL-SF™, by averaging the domain scores in respective three summary components. Summary scores range from 0 to 100; the higher the score, the better the HRQOL.

Potential factors associated with HRQOL were conceptualized through review of the literature. Along with many other factors; socio-demographic factors, kidney disease-related factors, psychological burden in terms of depression and psychological distress, the symptom burden due to CKD, and household cost to obtain health services; were identified as specific factors possibly associated with HRQOL and were assessed using the methods described as follow.

### Assessment of depression and psychological distress

Patients were screened for depression and psychological distress by locally validated Sinhalese versions of the Centre for Epidemiologic Studies Depression Scale, CES-D [19] and GHQ-12 [20], respectively. CES-D instrument has 20 items and out of the total score of 60, a score more than 15 is indicative of depression in the local setting with a sensitivity of 84% and a specificity of 92%. GHQ-12 has a total possible score of 12. A cut-off value of two or more is recommended to identify psychological distress with a sensitivity of 74% and a specificity of 71% [20].

### Assessment of symptom burden of CKD patients

CKD Symptom Index – Sri Lanka (CKDSI-Sri Lanka), which has been locally developed and validated, was used to assess the symptom burden. CKDSI-Sri Lanka was confirmed as having an excellent construct validity and test/re-test reliability [21]. The CKDSI –Sri Lanka consists of a checklist of 25 symptoms. Each one requires a response of ‘No’ if the patient did not experience the particular symptom during the seven days prior to the time of inquiry and the response ‘Yes’ if he/she did experience it during that time period. If the response is positive, the patient is then asked to rate the severity of the symptom on a 5-point Likert scale. In assessing the symptom burden, the severity rate of each symptom of 1 to 5, was treated as a score. Those who did not experience the symptom were given a score of zero. The symptom burden score for each respondent was the sum of the symptom severity scores for each of the symptoms included in the CKDSI-Sri Lanka. The possible score ranged from zero to 125.

### Assessment of household costs of CKD patients

Questions to assess the household cost were developed by reviewing literature and from inputs from CKD patients and caregivers. The instrument was pre-tested in Polonnaruwa district, a rural district in NCP [22]. Total household costs for the patient were calculated by assessing expenditures incurred for hospital admissions, dialysis and clinic visits; including the cost of transport, food, drugs, investigations, medical equipment and expenses for accompanying persons. Furthermore, expenditures on private medical consultations for treatment by Western medical practitioners, Ayurvedic medical practitioners or any other type of medical practitioners and religious rituals were also included.

### Survey administration and data collection

After patient enrolment, the data collector collected basic demographic data from the patient and administered the instruments; KDQOL-SF, CES-D, GHQ-12, CKDSI-Sri Lanka. Demographic data collected were age, gender, comorbidities status and employment status. Clinical, biochemical and treatment-related information was extracted from the patient’s personal medical record. The latest available serum creatinine value within three months of data collection was used to calculate the eGFR of the patient. The Modification of Diet in Renal Disease equation was used for this purpose.

Data collection has been done by five Public Health Inspectors working in the CKD unit in the North Central Province who had experience in functioning as data collectors for many local and international studies done among CKD patients. Data collection was mostly done on weekdays considering the fact that most of the study units were expected to be at home, since most are employed in the informal sector. Initially the eligibility of the selected study participants was assessed by the data collectors and if found eligible they were informed the purpose of the study and were invited to participate in the study. Further, the voluntary nature of participation and the opportunity to decline participation at any time of the survey, in spite of having agreed to participate was emphasized to them. When the selected person who lived in the house was not available at the time of visiting the house, additional two visits were done to the house at times that he/she was expected to be available. In cases where the selected study unit could not be contacted even after three visits, the study unit was considered as a non-respondent.

In instances where the selected study unit was found to be deceased or not eligible to be included in the study, the principal investigator conducted the process of random sampling described above and selected another study unit to be included.

### Statistical analysis

Statistical analysis was done by using SPSS version 20.0. The summary component scores of KDQOL-SF was non-normally distributed indicating a skewed distribution. Thus non-parametric tests were used in the bivariate analysis (Mann-Whitney U test and Spearman’s r correlation) when determining the association between kidney, physical and mental summary components and some selected factors. A *p* value of less than 0.05 was considered statistically significant. The following variables were analyzed with the each of the summary components; age, gender, education level, income status, employment status, the presence of comorbidities, CKD stage, symptom burden, presence of depression and Psychological distress. In order to explore how each socio demographic, as well as disease related characteristics, influence the HRQOL when the effects of other factors are controlled, multiple linear regression analysis was performed. Only the variables which had probability value of less than 0.2 in the univatiate linear regression analysis were included as independent variables in the multiple linear regression analysis. Normality of residuals was assessed using normal P-P plot while homoscedasticity was assessed using a scatter plot. The residuals were normally distributed and there was no evidence of heteroscedasticity. Evidence of outliers was assessed by Cook’s distance score and a score of less than ‘one’ was considered appropriate. Multi-collinearity was assessed using the Variance Inflation Factor (VIF), which should be less than ‘four’. The Cook’s distance was 0.026 and the VIF score was less than two.

## Results

Out of 1174 participants selected to be included in the study, 95 (8.1%) did not participate in the study giving a response rate of 91.9%. Of the 95 non-respondents, 69 (5.9%) were not contactable even after three consecutive visits and 16 (1.4%) didn’t consent to participate for the study. Ten (0.8%) completed less than half of the questionnaire due to various disturbances to the interview.

The mean age of the study population was 58.3 years (SD 10.7). There was a preponderance of males among the study population (63.1%, *N* = 681). The majority of participants was in the later stages, stage 4 or beyond, of CKD (*n* = 820; 75.9%). The 38 participants (3.5%), with stage 5 of the disease and were undergoing dialysis, were on haemodialysis (Table 1). Chronic Kidney Disease of Unknown origin was the cause for the CKD in most of the study population (*n* = 471; 43.7%). The second most common was hypertension (*n* = 347; *n* = 32.2%). The symptom burden score was assessed using CKDSI-Sri Lanka instrument and the median symptom burden score was 35.0 (IQR 20.0–49.0) while the mean score was 35.8 (SD 20.0). The median total monthly household income for the total study population was Rs.4000 (IQR 3000–7000), and the mean income was 6755.9 (SD 7492).

**Table 1:** Socio Demographic characteristics

Feature	Frequency (***N*** = 1079)	Percentage
Age categories		
18–40	50	4.6
41–60	558	51.7
> 60	471	43.7
Gender		
Male	681	63.1
Female	398	36.9
CKD stage		
Early stages	259	24.0
Stage IV	629	58.3
Stage V	153	14.2
Dialysis	38	3.5
Comorbidities		
Negative	308	28.5
Positive	771	71.5
Employment status		
Not employed	701	65.0
Employed	378	35.0
Depression		
Negative	375	34.8
Positive	704	65.2
Psychological distress		
Negative	267	24.7
Positive	812	75.3

In the KDSC, symptom/problem domain (72.7; IQR 58.3–81.8), effects of kidney disease (75.0; IQR 59.3–87.5) and hospital staff encouragement (75.0; IQR 75.0–75.0) domains had median scores above 70.0 while the burden of kidney disease had the lowest median score (31.2; IQR 25.0–43.7). Median scores of all the four domains in the PCS had the scores below 50.0. The highest median score was for the pain domain (42.5; IQR 20.0–50.0) while the lowest was for the role-physical (0.0; IQR 22.5–45.0). Except the domain role-emotion (0.0; IQR 25.0–62.5), all the other domains in the MCS, had their median scores 50.0 or close to 50.0 (Table 2).

**Table 2:** Distribution of the QOL Domain Scores of the Study Population as Measured By KDQOL SF™

Domains	Median	IQR	Mean	SD	Range
**Kidney Disease Specific Component**					
Symptom/problem domain	72.7	58.3–81.8	69.0	18.0	2.1–100.0
Effects of kidney disease	75.0	59.3–87.5	73.5	19.0	6.2–100.0
Burden of kidney disease	31.2	25.0–43.7	36.8	16.1	0.0–100.0
Work status	50.0	50.0–50.0	50.5	10.4	0.0–100.0
Cognitive function	66.6	53.3–80.0	65.5	17.1	0.0–100.0
Quality of social interaction	60.0	53.3–60.0	57.9	11.8	0.0–100.0
Sexual function (*n* = 625)	50.0	25.0–100.0	53.4	36.7	0.0–100.0
Sleep	52.5	40.0–62.5	52.6	15.8	10.0–97.5
Social support	66.6	33.3–100.0	66.1	25.9	0.0–100.0
Hospital staff encouragement	75.0	75.0–75.0	71.6	14.2	25.0–100.0
Patient satisfaction	50.0	50.0–50.0	47.4	11.3	16.7–83.3
**Overall Health**	50.0	40.0–60.0	50.8	17.2	0.0–100.0
**Physical Component Domains**					
Physical functioning	40.0	0.0–50.0	41.3	24.0	0.0–100.0
Role – physical	0.0	22.5–45.0	25.7	42.3	0.0–100.0
Pain	42.5	20.0–50.0	36.2	17.0	0.0–100.0
General health	30.0	44.0–52.0	37.6	19.6	5.0–90.0
**Mental Component Domains**					
Emotional well-being	48.0	0.0–33.3	48.1	9.7	8.0–84.0
Role – emotional	0.0	25.0–62.5	23.1	32.8	0.0–100.0
Social function	50.0	40.0–50.0	42.1	22.4	0.0–100.0
Energy/fatigue	50.0	25.0–75.0	44.8	12.2	0.0–85.0
**Change in Health**	50.0	25.0–75.0	52.10	29.9	0.0–100.0

Kidney Disease Summary Component score (KDSC) (58.4; IQR 54.2–63.4), was higher than the summary scores of Physical Component Summary Score (PCS) (35.0; IQR 26.2–41.9) and Mental Component Summary Score (MCS) (58.4; IQR 54.2–63.4) (Table 3). Both KDSC (*r* = − 0.066) and PCS (*r* = − 0.082) scores showed a slight, but statistically significant, negative correlation with the age. Even though strength of association was slight (*r* = 0.086), KDSC and MCS scores had significant (*p* < 0.05) positive correlation with the education status. The income was significantly positively correlated with all three summary components while the CKD stage was significantly negatively correlated with the same. Both males and females had the median scores equal (*p* > 0.05) in all the three summary scores. Currently being employed, screening positive for both depression and psychological distress were significantly (*p* < 0.001) associated with low HRQOL in all the summary scores (Table 4).

**Table 3:** Correlation between QOL domain scores and some selected factors

Domain	Mean (SD)	Median	IQR	Age^b^	Education level^b^	Income^b^	CKD stage^b^	Symptom burden^b^
Kidney Disease Summary Component	58.7 (7.7)	58.4	54.2–63.4	−0.066^a^	0.086^a^	0.180^a^	−0.122^a^	0.066^a^
Physical Component Summary Score	35.5 (15.0)	35.0	26.2–41.9	− 0.082^a^	0.052	0.152^a^	− 0.327^a^	0.101^a^
Mental Component Summary Score	39.6 (12.3)	39.0	34.6–42.8	− 0.047	0.077^a^	0.155^a^	− 0.346^a^	0.038

^a^Significant at the 0.05 level

^b^Association tested by Spearman correlation

**Table 4:** Association between kidney, physical and mental summary components and some selected factors

Characteristic	Kidney Disease Summary Component			Physical Component Summary Score			Mental Component Summary Score		
	Median	IQR	***p*** value^**a**^	Median	IQR	***p*** value^**a**^	Median	IQR	***p*** value^**a**^
**Gender**									
Male	58.5	54.2–63.7	0.470	35.0	26.2–40.9	0.443	38.8	34.8–42.2	0.470
Female	58.2	54.3–63.1		36.2	25.0–43.1		39.1	33.5–43.7	
**Employment**									
Employed	60.4	56.0–65.0	< 0.001	36.3	30.0–45.6	< 0.001	39.5	36.2–43.3	< 0.001
Not employed	57.6	53.3–62.5		33.7	23.7–40.6		38.4	32.3–42.4	
**Comorbidities**									
Present	58.2	53.8–62.8	0.002	35.0	25.0–42.5	0.539	39.0	34.0–43.0	0.515
Absent	59.6	55.3–65.2		35.0	27.5–41.1		39.1	35.5–42.5	
**Depression**									
Positive	56.4	52.6–60.6	< 0.001	33.7	23.7–40.0	< 0.001	37.9	30.5–41.3	< 0.001
Negative	63.3	58.5–67.9		36.2	30.0–48.1		40.1	37.1–46.1	
**Psychological distress**									
Positive	57.2	53.3–61.9	< 0.001	33.7	23.7–39.4	< 0.001	38.2	32.1–41.3	< 0.001
Negative	63.5	57.8–68.5		41.2	32.5–57.5		41.3	37.7–52.8	

^a^Association tested by Mann-Whitney U test

In order to explore how each socio demographic, as well as disease related characteristics, influenced the HRQOL of CKD when the effects of other factors are controlled, multiple linear regression analysis was performed. Age and sex were not significant predictors of either KDSC, PCS or MCS (*p* > 0.05). Education level was a significant predictor (*p* < 0.05) of higher scores of both KDSC (β = 0.535) and MCS (β = 0.870), while the being employed was a significantly associated with higher scores of both KDSC (β = 1.394) and PCS (β = 1.966). Advanced stages of CKD, being positive for depression and being positive for psychological distress were significantly associated with low HRQOL in all the three summary component scores (Table 5).

**Table 5:** Independent predictors of QOL using multiple linear regression

	Kidney Component Summary Score		Physical Component Summary Score		Mental Component Summary Score	
	Beta coefficient	95% CI	Beta coefficient	95% CI	Beta coefficient	95% CI
**Age**	0.02	−0.02 to 0.059	−0.005	− 0.08 to 0.07	NA	
**Gender**						
Male	NA		NA		NA	
Female	NA		NA		NA	
**Education level**	0.52^a^	0.08 to 0.95	0.632	−0.22 to 1.49	0.84^a^	0.16 to 1.53
**Employment**						
Employed	1.27^a^	0.30 to 2.23	1.94^a^	0.05 to 3.83	1.04	−0.45 to 2.53
Not employed	1		1		1	
**Income (in thousands)**	0.05	−0.08 to 0.07	0.15^a^	0.03 to 0.27	0.12^a^	0.03 to 0.22
**Comorbidities**						
Present	−0.38	−1.27 to 0.52	NA		NA	
Absent	1		NA		NA	
**CKD stage**	−1.38^a^	−1.92 to −0.82	−6.37^a^	−7.46 to −5.28	−6.31^a^	−7.19 to −5.44
**Depression**						
Positive	−5.31^a^	−6.25 to −4.37	−2.16^a^	−4.01 to −0.31	−3.35^a^	−4.8 to − 1.8
Negative	1		1		1	
**Psychological distress**						
Positive	−3.31^a^	−4.34 to −2.28	−9.30^a^	−11.3 to − 7.2	− 6.02^a^	− 7.6 to −4.3
Negative	1		1		1	
**Symptom burden**	NA		0.06^a^	0.02 to 0.10	NA	

^a^Significant at the 0.05 level

NA – The variable is not significant in the univariate linear regression, thus not included in the multiple linear regression

## Discussion

In the present study, the HRQOL of the study population was assessed using a locally validated KDQOL SFT^M^. This is the first study in Sri Lanka which assessed the HRQOL of a community severely crippled by CKDu, and revealed that HRQOL among these population was severely compromised.

The KDQOL-SF™ is a popular tool that has been used to assess HRQOL among CKD patients by researchers the world over [9, 23]. The KDSC, PCS and MCS in the present study were 58.7 (SD 7.7), 35.5 (SD 15.0) and 39.6 (SD 12.3) respectively, indicating that the HRQOL of these CKD patients in the rural community of Anuradhapura is poor. According to Mujais et al. (2009) who assessed the HRQOL of CKD patients in North America the mean KDSC, PCS and MCS scores were 74.6 (SD 13.6), 39.5 (10.6) and 49.8 (10.4) respectively. Except the KDSC, the scores of PCS and MC in the above study was almost similar to the findings of our study indicating that the HRQOL of CKD patients is compromised even in superior socio-economic and health contexts. This was further reiterate by the findings of Yusop et al. (2013) [23]. He assessed HRQOL of the CKD patients in Malaysia and the PCS (39.6; SD 8.6) and the MCS (45.0; SD 8.6) were almost similar to our findings.

Over the last few decades HRQOL strategies were mainly focusing on cancer patients, but studies have shown that patients with other life threatening conditions also experience a similar degree of effect to HRQOL [24]. Evidence indicate that both physical and mental distress experienced by patients dying from renal failure, could be more distressing than cancer [25]. Chronic kidney disease patients suffer from both physical and psychological symptoms which may emerge at different stages during the course of the disease [26]. Jang et al. (2019) assessed the HRQOL of patients with severe chronic obstructive pulmonary disease (COPD) using SF 36 study instrument and the PCS and MCS were 56.7 and 69.5 indicating that the HRQOL of CKD patients is much worse compared to COPD [27]. Similarly two international studies done to assess the HRQOL of stroke [28] and chronic heart failure [29] revealed that the stroke patients had relatively low HRQOL and chronic heart failure patients had a similar HRQOL when compared to the results of our study participants. Mahesh et al., (2017) conducted three separate studies to assess the HRQOL of stroke [28], myocardial infarction [30] and COPD [31] patients in Sri Lanka using SF 36. All eight domain scores of PCS and MCS among myocardial infarction patients were superior to the respective domain scores in our study. Furthermore, among COPD patients, except the general health, physical functioning, energy/fatigue and emotional well-being, the other four domains had superior domain scores compared to CKD. Compared to the CKD patients, general health, physical functioning, emotional well-being and social functioning domain scores were low among the stroke patients. This indicate that compared to the three conditions studied by Mahesh et al., stroke, COPD and myocardial infarction, CKD patients had poor HRQOL in relations to pain and physical and emotional role limitations.

In the present study the KDSC was significantly higher compared to the PCS and MCS. A similar finding was evident in the multi country study done by Mujais et al., (2009), where the relatively higher scores were obtained for the domains of the Kidney Disease Specific Component [9]. It could be due to the fact that the scores of the disease specific component of the study tool is only sensitive to the severity of the disease, whereas the generic component scores of the study tool (physical and mental component) are sensitive not only to the severity of the disease but also to multiple other socio-economic factors, which are unfavorable for the CKD patients in a rural community with a poor socio-economical background such as the Anuradhapura district.

It was surprising to note that in the present study, the gender of the patients was not a significantly predictor of HRQOL. This was contrary to the findings of Mujais et al. (2009) where female gender was a significant predictor of low HRQOL [9]. Unemployment due to the disease, difficulty in engaging in the current occupation and inability to perform the role expected from society, is more common among males in the local setting, which could lead to poor HRQOL among male CKD patients in the study setting.

Higher education level, being employed and higher income were significant independent predictors of higher summary component scores. This indicate that in general, the improvement of socio-economic and educational aspects is associated with better quality of life among these patients. Further, social acceptance, being able to perform social responsibilities and self-esteem which are directly linked to the employment status, income and education status of an individual [32, 33] and they also could have contributed to the superior HRQOL of patients with this subset of the study population.

CKD stage was a significant independent predictor of scores in all three summary scores, but the magnitude of association was much higher in PCS and MCS, compared to the association between CKD stage and KDSC. One would expect the KDSC, which assess the kidney disease specific HRQL, to show the highest association with CKD stage compared to others as reported in several studies internationally [9, 34]. However, a similar finding to the current study has been observed in the study conducted by Perlman et al., (2005) and the authors have ascribed the results to the narrow range of glomerular filtration rate (GFR) in their study [35], which could be the reason in the current study as well.

The results indicate that the symptom burden significantly affects the HRQOL related to the physical and mental health status in CKD patients (PCS and MCS). This picture was further reinforced by the fact that depressed and psychologically distressed patients had significantly low HRQOL scores in all three summary scores. This finding has been reiterated in the global literature as well. Symptom burden [36] and mental health status [37] were stated as key determinants of HRQOL of CKD patients. This highlights an important finding of clinical importance, that is if the service providers to improve the HRQOL of patients, not only physical symptom alleviating measures but also measures to improve the mental health status of patients should also be considered.

Our study has some limitations. Firstly, the HRQOL assessment did not employ a control group due to feasibility and logistic constraints. Inclusion of a control group could have enabled comparison between the two groups to produce more meaningful interpretation. Secondly, some of the information related to HRQOL is considered to be sensitive in nature and the fact that this information was obtained utilizing an interviewer-administered questionnaire could have led to some under-reporting in the assessment of HRQOL, though many measures were taken to minimize this issue. Finally, The CKD stage in this study was calculated using secondary data. Biochemical assessment of study units was not economically feasible in the study.

## Conclusion

The HRQOL of the CKD patients in this rural Sri Lankan population was found to be poor. Higher education status and being employed were important predictors of higher HRQOL, while advanced stages of CKD, being positive for depression and being positive for psychological distress were significantly associated with low HRQOL. The study recommends numerous measures to improve the HRQOL of these patients. Periodical screening of the CKD patients for depression and psychological distress and measures to alleviate symptom burden seem to be important and practical aspects which can be implemented to improve the overall quality of life of these patients.

## Supplementary information

**Additional file 1.** Supplementary material Number of CKD patients selected from each MOH area for the study.

## Data Availability

The datasets used and/or analysed during the current study are available from the corresponding author on reasonable request.

## Abbreviations

CES-D: Center for Epidemiological Studies – Depression scale
CKD: Chronic Kidney Disease
CKDSI-Sri Lanka: Chronic Kidney Disease Symptom Index – Sri Lanka
COPD: Chronic Obstructive Pulmonary Disease
GFR: Glomerular Filtration Rate
GHQ-12: General Health Questionnaire 12
HRQOL: Health Related Quality of Life
IQR: Inter Quartile Range
KDQOL-SF: Kidney Disease Quality of Life – Short Form
KDSC: Kidney Disease Summary Component
MCS: Mental Component Summary
MOH: Medical Officer of Health
NCP: North Central Province
PCS: Physical Component Summary
SD: Standard Deviation
WHOQOL: World Health Organization Quality of Life Group
