# Supplementary material for: Health related quality of life in chronic kidney disease; a descriptive study in a rural Sri Lankan community affected by chronic kidney disease
Source: Health Qual Life Outcomes. 2020 Apr 23;18:106. doi: 10.1186/s12955-020-01369-1 (PMC7178581; doi:10.1186/s12955-020-01369-1)
Supplement: Supplementary file 1 — Additional file 1. Supplementary material Number of CKD patients selected from each MOH area for the study. [file 12955_2020_1369_MOESM1_ESM.pdf]

Supplementary material

<u>Number of CKD patients selected from each MOH area for the study</u>

		Total number of CKD patients recorded in the register	Proportion from each MOH area (%)	Number of CKD patients selected from each MOH area
1	Madawachchiya	2635	23.2	273
2	Padaviya	1337	11.8	138
3	Kabithigolawa	1053	9.3	109
4	Rambewa	937	8.3	97
5	Kahatagasdigiliya	823	7.3	85
6	Nuwaragampalatha Central	661	5.8	68
7	Horopothana	553	4.9	57
8	Galenbidunuwewa	552	4.9	57
9	Nuwaragam Palatha East	420	3.7	43
10	Thalawa	368	3.2	38
11	Mihinthale	325	2.9	34
12	Thambuttegama	312	2.7	32
13	Nochchiyagama	310	2.7	32
14	Kekirawa	297	2.6	31
15	Galnewa	180	1.6	19
16	Rajanganaya	179	1.6	19
17	Thirappane	171	1.5	18
18	Iplogama	130	1.1	13
19	Palagala	103	0.9	11
		**11346**	**100.0**	**1174**
